# Myocardial impairment in rheumatoid arthritis patients with normal left ventricular function: findings from speckle tracking echocardiography

**DOI:** 10.3389/fmed.2025.1530622

**Published:** 2025-05-02

**Authors:** Rim Dhahri, Mahfoudhi Houaida, Ismail Dergaa, Lobna Ben Ammar, Halil İbrahim Ceylan, Youssef Ben Abderrazek, Insaf Fenniche, Sarra Chenik, Wafa Fehri, Raul Ioan Muntean, Imène Gharsallah

**Affiliations:** ^1^Rheumatology Department, Military Hospital of Tunis, Faculty of Medicine of Tunis, University Tunis El Manar, Tunis, Tunisia; ^2^Department of Cardiology, Military Hospital of Tunis, Faculty of Medicine of Tunis, University Tunis El Manar, Tunis, Tunisia; ^3^High Institute of Sport and Physical Education of Ksar Said, University of Manouba, Manouba, Tunisia; ^4^Physical Education of Sports Teaching Department, Faculty of Sports Sciences, Ataturk University, Erzurum, Türkiye; ^5^Department of Physical Education and Sport, Faculty of Law and Social Sciences, University “1 Decembrie 1918” of Alba Iulia, Alba Iulia, Romania

**Keywords:** anemia, cardiac function, cardiovascular comorbidities, diabetes mellitus, hemodynamic monitoring, inflammatory biomarkers, left ventricular dysfunction, rheumatoid heart disease

## Abstract

**Background:**

Rheumatoid arthritis (RA) is a chronic inflammatory condition recognized for elevating cardiovascular morbidity and mortality, even in the absence of overt cardiovascular symptoms. Traditional echocardiogram frequently overlooks early myocardial failure, necessitating more sensitive imaging modalities, such as speckle tracking echocardiography (STE), to effectively diagnose subclinical left ventricular systolic dysfunction (LVSD). Timely identification of cardiac involvement is essential for reducing long-term cardiovascular risks in people with rheumatoid arthritis.

**Objectives:**

This study sought to (i) determine if STE can identify subclinical myocardial dysfunction in RA Patients with normal left ventricular function as assessed by transthoracic echocardiography and (ii) identify clinical and biological factors linked to this extra-articular manifestation.

**Methods:**

A total of 36 RA patients and 36 matching healthy controls were included. All subjects underwent standard transthoracic echocardiogram and speckle tracking STE to evaluate left ventricular function. Global longitudinal strain (GLS) was employed to identify subclinical left ventricular systolic dysfunction, with a GLS threshold of ≤ − 18% signifying LVSD. Clinical and biochemical variables, such as hemoglobin concentrations, diabetes mellitus, and disease activity (DAS28-CRP), were evaluated to determine their correlation with compromised myocardial strain.

**Results:**

RA patients had a significantly diminished GLS compared to healthy controls (18.99 ± 2.81% vs. 20.42 ± 1.33%, *p* = 0.015), notwithstanding a normal left ventricular ejection fraction (LVEF). Subclinical LVSD was detected in 33% of RA patients, but none of the control subjects exhibited this condition. Anemia was identified as the most significant independent predictor of diminished GLS (OR: 11.39, 95% CI: 1.57–82.89, *p* = 0.016), although diabetes mellitus and age exhibited associations with myocardial strain in univariate analysis. No substantial correlations were identified between GLS and disease activity (DAS28-CRP) or immunological markers (RF, anti-CCP).

**Conclusion:**

STE identified subclinical LVSD in a significant number of RA patients with normal LVEF, emphasizing its effectiveness in early cardiovascular risk assessment. Hemoglobin levels were a crucial predictor of subclinical LVSD, highlighting the necessity of thorough cardiovascular risk evaluations in RA, especially for individuals with anemia or other concomitant conditions. Incorporating STE into standard assessments may facilitate early interventions and enhance long-term cardiovascular outcomes for patients with RA.

## 1 Introduction

Rheumatoid arthritis (RA) is a chronic autoimmune disorder that predominantly impacts synovial joints, marked by ongoing inflammation and tissue destruction (1). In addition to its joint manifestations, RA exhibits systemic consequences that impact various organs, including the cardiovascular, pulmonary, renal, and ocular systems, thereby considerably influencing the morbidity and mortality linked to the disease (1).

Cardiovascular disease in RA is particularly alarming, as RA patients exhibit a heightened risk of heart failure (HF) and ischemic heart disease relative to the general population, with evidence indicating that cardiovascular complications may arise even in the absence of conventional risk factors (2, 3). RA is associated with an elevated risk of both fatal and non-fatal cardiovascular events, highlighting its impact on reducing life expectancy (2, 3).

In RA, heart failure frequently exhibits intact left ventricular ejection fraction (LVEF), however, its clinical manifestations are typically mild and correlate with elevated mortality rates compared to non-RA populations (4). Research has established similarities between the cardiovascular risk profiles of patients with RA and those with diabetes mellitus (DM), underscoring the necessity for focused cardiovascular risk assessment in RA patients (5, 6). Nonetheless, a consensus on the most effective technique for the early detection or prevention of preclinical cardiac involvement in RA patients has yet to be established (5, 6).

Speckle tracking echocardiography (STE) is a contemporary, non-invasive imaging technique that evaluates myocardial deformation (strain), offering a more sensitive assessment of left ventricular function than conventional transthoracic echocardiogram. In contrast to LVEF, which indicates overall systolic function, STE can identify subclinical left ventricular systolic dysfunction (LVSD) by assessing localized myocardial strain, even when LVEF is normal (7). STE is very effective in detecting early cardiac deterioration in RA patients (7).

Notwithstanding the increasing acknowledgment of cardiovascular involvement in RA, numerous gaps persist in the existing literature (8, 9). Although it is recognized that RA patients have a heightened risk of LVSD, research examining the efficacy of STE in identifying subclinical myocardial dysfunction in RA patients with normal LVEF remains few (8, 9). Moreover, variables like anemia, disease duration, and conventional cardiovascular risk factors (e.g., diabetes mellitus and hypertension) have shown contradictory associations with subclinical LVSD in RA patients (8, 9). Further investigation is required into the clinical and molecular markers that may predispose RA patients to early heart dysfunction, as existing research yields inconclusive results. Moreover, limited research has investigated the possible effects of disease-modifying antirheumatic medications (DMARDs) on subclinical cardiac dysfunction, especially concerning alterations in myocardial strain (10). Rectifying these deficiencies is essential for enhancing cardiovascular risk assessment and management in patients with RA.

Thus, this study aimed to (i) find out whether STE may recognize subclinical myocardial impairment in RA patients with normal left ventricular function in transthoracic echocardiography and (ii) identify clinical and biological factors associated with this extra-articular manifestation.

## 2 Methods

### 2.1 Study design and participants

This study was a monocentric, cross-sectional study carried out from March 2019 to September 2019. The study population consisted of 36 patients with RA who fulfilled the 2010 American College of Rheumatology/European Alliance of Associations for Rheumatology (ACR/EULAR) classification criteria for RA and were above 18 years of age. For comparison, these patients were paired with 36 healthy control participants according to age, sex, and body mass index (BMI). Matching was crucial to eliminate potential confounding variables associated with demographic parameters. Individuals with a history of cardiomyopathy, coronary artery disease, arrhythmia, cardiac surgery, valve malfunction, or those who had received chemotherapy were excluded. Furthermore, those exhibiting inadequate echocardiographic imaging, substantial valvular abnormalities, left ventricular systolic failure (characterized by LVEF < 55%), or any wall motion abnormalities identified using transthoracic echocardiography were likewise excluded.

### 2.2 Ethical approval

All participants provided informed consent before enrollment, and the study protocol was reviewed and approved by the Ethics Committee of the Military Hospital of Tunis on (15.06.2020) under the reference (63/2020/CLPP/Military Hospital of Tunis).

### 2.3 Data collection

#### 2.3.1 Demographic and clinical data

A highly qualified physician gathered demographic and clinical data via patient interviews and physical examinations. The obtained demographic data included sex, age, smoking status, and BMI. The BMI was determined utilizing a KERN MPE 250K100PM floor scale, guaranteeing precision and uniformity in measurements (11).

The disease-specific characteristics of the RA group were meticulously recorded. This included the age at diagnosis, the interval between symptom onset and diagnosis, disease duration, the occurrence of joint erosion (evaluated using plain radiography, ultrasonography, or magnetic resonance imaging), and the immunological profile. The immunological profile was evaluated using rheumatoid factor (RF), anti-citrullinated protein antibodies (anti-CCP), and antinuclear antibodies (ANA). Data regarding the use of corticosteroids and disease-modifying anti-rheumatic Drugs (DMARDs) was also collected. Disease activity was assessed with the Disease Activity Score in 28 joints with C-reactive protein (DAS28-CRP) (12).

#### 2.3.2 Laboratory investigations

Complete blood cell count (CBC) and C-reactive protein (CRP) tests were conducted to evaluate the patients’ systemic inflammatory status and hematologic profile. Anemia was defined according to World Health Organization (WHO) rules, with hemoglobin levels being abnormal if they are below 12 g/dL in women or below 13 g/dL in men (13).

#### 2.3.3 Echocardiographic assessments

##### 2.3.3.1 Conventional transthoracic echocardiography

All echocardiographic assessments were conducted by a single cardiologist, who remained unaware of the patient’s clinical information. The transthoracic echocardiogram was performed on a General Electric Vivid 7 echocardiography system with a 3.5 MHz transducer. The left ventricular ejection fraction (LVEF) was assessed using Simpson’s biplane method, while conventional echocardiographic parameters included left ventricular end-diastolic diameter (LVEDD), left ventricular end-systolic diameter (LVESD), interventricular septal thickness (IVST), and posterior wall thickness (PWT). Pulsed-wave Doppler was employed to evaluate transmittal flow, with the E/A ratio computed to estimate diastolic function. Color-coded tissue Doppler imaging (TDI) was employed to quantify the systolic mitral annulus velocity (S′) and early diastolic mitral annular velocity (E’) (11).

##### 2.3.3.2 Speckle tracking echocardiography (STE)

STE was utilized to assess global longitudinal strain (GLS), providing a more sensitive evaluation of left ventricular function. The speckle-tracking software monitored myocardial deformation by examining the movement of speckles throughout the cardiac cycle in gray-scale echocardiographic images. Three cardiac cycles were recorded in Digital Imaging and Communications in Medicine (DICOM) format, including apical four-chamber, two-chamber, and long-axis perspectives. GLS was determined as the mean of segmental stresses utilizing GE Healthcare EchoPAC software. Subclinical LVSD is characterized by a GLS of ≤ − 18%, following the standards set by the American Society of Echocardiography (ASE) and the European Association of Cardiovascular Imaging (EACVI) (11). All strain data were presented as absolute values to eliminate ambiguity concerning positive or negative strain variations.

## 3 Statistical analysis

All statistical analyses were performed with SPSS version 25.0. Continuous variables were represented as mean values (± standard deviation) for normally distributed data, or as medians with interquartile ranges (IQR) for non-normally distributed variables. Categorical variables were represented as percentages. Parametric and non-parametric tests were utilized as appropriate, including the student’s t-test for independent samples and the Mann–Whitney U test for non-normally distributed data. Chi-square or Fisher’s exact tests were employed for the comparison of categorical variables.

The associations among clinical, biological, and echocardiographic variables were evaluated using Pearson’s correlation coefficient for continuous variables, and point-biserial correlation for dichotomous and continuous data. Multivariate logistic regression analyses were used to identify independent variables linked to subclinical LVSD, incorporating variables that were significant at *p* < 0.05 in the univariate study into the multivariate model. The Receiver Operating Characteristic Recurve was employed to evaluate the predictive significance of hemoglobin levels, age, and E/A ratio for subclinical LVSD. All statistical tests were conducted as two-tailed, with a significance level set at *p* < 0.05.

## 4 Results

The study population involved 36 RA patients and 36 healthy controls, matched by age, sex, and BMI. The baseline characteristics and comorbidities of the two groups are summarized in Table 1. The average age of the RA group was 51.69 ± 12.14 years, with 75% of participants being female. Comorbid disorders such as diabetes mellitus, hypertension, dyslipidemia, and active smoking were more common in the RA group compared to the control group. Diabetes mellitus was observed in 25% of RA patients, and hypertension in 17%, both of which were absent in the control group, with significant *p*-values (*p* = 0.002 and *p* = 0.025, respectively).

**Table 1:** Baseline demographics and comorbidities of rheumatoid arthritis and control group.

Variables	RA group *N* = 36	Control group *N* = 36	*P*
Age (year)	51.69 ± 12.14	51.92 ± 11.94	0.94
Female (%)	27 (75%)	27 (75%)	0.99
BMI (kg/m^2^)	28.17 ± 4.27	27.10 ± 3.35	0.25
Obesity	8 (24%)*	10 (28%)	0.74
Diabetes mellitus	9 (25%)	0	0.002
Hypertension	6 (17%)	0	0.025
Dyslipidemia	6 (17%)	0	0.025
Hypothyroidism	2 (6%)	0	0.49
Active smokers	6 (17%)	0	0.025

BMI, Body mass index; RA, Rheumatoid arthritis; * The BMI of three patients could not be assessed.

### 4.1 Characteristics of disease in RA patients

Table 2 presents the clinical, biochemical, and immunological characteristics of the RA group. The average disease duration was 12.14 ± 6.97 years, with joint erosion observed in 78% of the patients. The average DAS28-CRP was 4.01 ± 1.43, signifying moderate to high disease activity in most cases. In terms of immunological profiles, 67% of RA (RA) patients tested positive for rheumatoid factor (RF), while 72% tested positive for anti-citrullinated protein antibodies (anti-CCP). A significant percentage of the RA group (36%) demonstrated anemia, as per WHO standards.

**Table 2:** Clinical, biological and immunological characteristics of rheumatoid arthritis group.

Variables		RA group *N* = 36
Age at diagnosis (year)		43.31 ± 12.72
Disease duration (year)		12.14 ± 6.97
Joint erosion (%)		78
DAS28-CRP		4.01 ± 1.43
Immunological profile	RF (%)	67
	Anti-CCP (%)	72
	ANA (%)	17
Biochemical findings	CRP (mg/L)	7 (17–7)
	Hb (g/dl)	12.2 (13–11)
	WBC (10^3^/μl)	8.1 (10–5.8)
	Neutrophils (10^3^/μl)	4.3 (5.8–3)
	Lymphocytes (10^3^/μl)	1.9 (2.9–1.6)
	Platelets (10^3^/μl)	295 (375–214)
Treatments	MTX and CS (%)	61
	CS (%)	81
	MTX (%)	75
	Leflunomide (%)	6
	Sulfasalazine (%)	3
	Il-6 inhibitors (%)	17
	TNF inhibitors (%)	11
	CD20 blockers (%)	6

ANA, Antinuclear antibody; anti-CCP, Anti-citrullinated protein antibodies; B-DMARDs, Biologic disease-modifying anti-rheumatic drugs; CRP, C-reactive protein; CS, Corticosteroids; IL-6 inhibitors, Interleukin 6 inhibitors; MTX, Methotrexate; RA, Rheumatoid arthritis; RF, Rheumatoid factor; TNF inhibitors, Tumor necrosis factor inhibitors; WBC, White blood cell.

### 4.2 Conventional echocardiographic findings

Table 3 indicates that conventional echocardiography showed no significant differences in left ventricular dimensions or systolic performance between the RA and control groups. The left ventricular ejection fraction (LVEF) was normal in both groups, with the RA group exhibiting a mean LVEF of 69.86 ± 5.11% and the control group 68.28 ± 4.77% (*p* = 0.14). Tissue Doppler imaging (TDI) similarly revealed no significant variations in systolic mitral annulus velocity (S′) or diastolic function metrics, such as the E/A ratio and E/E’. Grade I diastolic dysfunction was identified in 19% of RA patients and 14% of controls, with no statistically significant differences between the groups (*p* = 0.53).

**Table 3:** Conventional echocardiography and tissue Doppler imaging findings.

Echocardiographic parameters	RA group *N* = 36	Control group *N* = 36	*P*
LVEDD (mm)	48.18 ± 3.79	48.22 ± 4.28	0.97
LVESD (mm)	27.41 ± 3.44	28.88 ± 4.53	0.13
IVST (mm)	7.87 ± 1.28	8.22 ± 1.17	0.16
PWT (mm)	7.75 ± 1.18	7.94 ± 1.17	0.65
LVM (g)	124.17 ± 22.4	131.67 ± 35	0.55
ILVM (g/m^2^)	70.31 ± 13.21	72.59 ± 19.28	0.57
LVEF (%)	69.86 ± 5.11	68.28 ± 4.77	0.14
S′ (cm/s)	8.59 ± 1.6	8.7 ± 1.17	0.44
E/A	1.09 ± 0.35	1.13 ± 0.29	0.69
E/E	6.16 ± 1.56	6.52 ± 1.32	0.13
Grade I diastolic dysfunction (N)	7 (19%)	5 (14%)	0.53

A, Transmitral late filling velocity; E, Transmitral early filling velocity; E/E’, Ratio of transmitral early filling velocity to the early diastolic mitral annular velocity; ILVM, Left ventricular mass indexed to body surface area; LVEDD, Left ventricular end-diastolic diameter; IVST, Interventricular septal thickness at end-diastole; LVEF, Left ventricular ejection fraction; LVESD, Left ventricular end-systolic diameter; PWT, Posterior wall thickness at end-diastole; RA, Rheumatoid arthritis; S′, Systolic mitral annulus velocity.

### 4.3 Speckle tracking echocardiography (STE) and subclinical myocardial dysfunction

STE demonstrated significant differences in GLS between the RA and control cohorts. RA patients demonstrated a significantly lower GLS of −18.99 ± 2.81% in contrast to the control group, which had a GLS of −20.42 ± 1.33% (*p* = 0.015), suggesting early myocardial dysfunction despite preserved left ventricular ejection fraction (LVEF) (Table 4). Subclinical LVSD, characterized by a GLS of ≤ − 18%, was identified in 33% of RA patients, but none of the healthy controls satisfied the criteria for subclinical LVSD.

**Table 4:** Correlations between baseline demographics, comorbidities, RA characteristics, and GLS in the RA group.

RA group		Pearson’s r	p
Age		−0.365	0.029
Male		0.027	0.88
BMI		0.076	0.67
Comorbidities	Obesity	0.081	0.65
	Diabetes Mellitus	−0.540	0.001
	Hypertension	−0.024	0.89
	Dysthyroidism	−0.118	0.49
	Dyslipidemia	0.167	0.33
	Active smoking	0.046	0.79
Age of diagnosis		−0.305	0.07
Duration of RA		−0.173	0.31
Joint erosion		0.069	0.69
DAS28-CRP		0.233	0.17
Immunological profile	RF	−0.194	0.26
	Anti-CCP	−0.043	0.80
	ANA	−0.093	0.59
Biochemical findings	Anemia	−0.368	0.027
	Hemoglobin	0.224	0.21
	WBC	−0.177	0.32
	Neutrophils	−0.106	0.56
	Lymphocytes	−0.109	0.55
	Platelets	−0.289	0.11
	CRP	0.157	0.36
Treatments	MTX and CS	−0.048	0.78
	MTX	0.036	0.83
	Weekly intake of MTX	−0.051	0.77
	CS	0.027	0.87
	Daily intake of CS	−0.010	0.95
	B-DMARDs	0.052	0.77
	TNF-inhibitors	0.137	0.42
	IL6-inhibitors	−0.177	0.30

ANA, Antinuclear antibody; anti-CCP, Anti-citrullinated protein antibodies; BMI: Body mass index; B-DMARDs, Biologic disease-modifying anti-rheumatic drugs; CRP, C-reactive protein; CS, Corticosteroids; GLS: Global longitudinal Strain; IL-6 inhibitors: Interleukin 6 inhibitors; MTX: Methotrexate; Obesity: BMI > 30 kg/m^2^; RA: Rheumatoid arthritis; RF: Rheumatoid factor; TNF inhibitors: Tumor necrosis factor inhibitors; WBC: White blood cell.

### 4.4 Associations between GLS and subclinical left ventricular systolic dysfunction (LVSD)

Univariate analysis revealed that anemia (*r* = −0.368, *p* = 0.027), age (*r* = −0.365, *p* = 0.029), and diabetes mellitus (*r* = −0.540, *p* = 0.001) were significantly correlated with poorer GLS in RA patients. Furthermore, the E/A ratio (*r* = 0.351, *p* = 0.036) exhibited a favorable correlation with GLS (Table 5). In contrast, disease activity metrics, including DAS28-CRP, and disease duration had no significant correlation with GLS, indicating that alternative factors, notably comorbidities may influence subclinical myocardial dysfunction.

**Table 5:** Correlations between conventional echocardiographic parameters and GLS among RA group.

RA group	Pearson’s r	*p*
LVEDD	0.026	0.88
LVESD	−0.015	0.93
IVST	−0.214	0.21
PWT	−0.226	0.19
LVM	−0.214	0.21
ILVM	−0.316	0.07
LVEF	0.022	0.90
S′	0.119	0.49
E/A	0.351	0.036
E/E’	0.063	0.72
Grade I diastolic dysfunction	0.313	0.06

A, Transmitral late filling velocity; E, Transmitral early filling velocity; E/E’, Ratio of transmitral early filling velocity to the early diastolic mitral annular velocity; GLS, Global longitudinal Strain; ILVM, Left ventricular mass indexed to body surface area; LVEDD: Left ventricular end-diastolic diameter; IVST: Interventricular septal thickness at end-diastole; LVEF: Left ventricular ejection fraction; LVESD: Left ventricular end-systolic diameter; PWT: Posterior wall thickness at end-diastole; RA: Rheumatoid arthritis; S′: Systolic mitral annulus velocity.

### 4.5 Determinants of subclinical left ventricular systolic dysfunction (LVSD)

In the multivariate logistic regression analysis (Table 6), anemia was identified as the sole independent predictor of subclinical LVSD, exhibiting an odds ratio (OR) of 11.39 (95% CI: 1.57–82.89, *p* = 0.016). Diabetes mellitus, although notable in univariate analysis, lost significance in the multivariate model (OR = 4.44, 95% CI: 0.67–29.29, *p* = 0.12). Hemoglobin levels appeared as the most reliable predictor of subclinical LVSD, as indicated by ROC curve analysis (AUC = 0.752, 95% CI: 0.577–0.927, *p* = 0.02), surpassing both age and the E/A ratio (Table 7).

**Table 6:** Multivariate regression analysis of parameters associated with subclinical left ventricular systolic dysfunction.

RA group	OR	95% CI	*p*
Anemia	11.39	1.57–82.89	0.016
Diabetes mellitus	4.44	0.67–29.29	0.12
E/A	0.105	0.01–1.94	0.13

A, Transmitral late filling velocity; E, Transmitral early filling velocity; RA, Rheumatoid arthritis.

**Table 7:** ROC curves analysis for prediction of subclinical left ventricular systolic dysfunction.

Variables	AUC	95% CI	*p*
Hemoglobin	0.752	0.577–0.927	0.02
Age	0.583	0.375–0.792	0.42
E/A	0.611	0.415–0.808	0.28

A, Transmitral late filling velocity; AUC, Area under the curve; CI, Confidence interval; E, Transmitral early filling velocity; ROC, Receiver operating characteristic.

## 5 Discussion

This study aimed to (i) ascertain whether STE could detect subclinical myocardial impairment in RA patients exhibiting normal left ventricular function in transthoracic echocardiography and (ii) identify clinical and biological factors associated with this extra-articular manifestation. Our findings indicated that a substantial percentage of RA patients, despite exhibiting a normal left ventricular ejection fraction (LVEF) on conventional echocardiography, demonstrated subclinical LVSD as identified by STE. These findings highlight the significance of early identification of myocardial impairment in RA since subclinical cardiac dysfunction might develop even without apparent cardiovascular symptoms, potentially resulting in detrimental cardiovascular consequences if inadequately managed.

### 5.1 Conventional echocardiographic findings

This study found that conventional echocardiography did not demonstrate significant changes in parameters including left ventricular end-diastolic diameter (LVEDD), left ventricular end-systolic diameter (LVESD), or diastolic function, as indicated by the E/A ratio. Both cohorts, comprising the RA and control populations, exhibited preserved LVEF (RA: 69.86 ± 5.11%, control: 68.28 ± 4.77%). This aligns with other research suggesting that conventional echocardiographic parameters often remain within normal limits in RA patients, despite underlying myocardial dysfunction. Indexed left ventricular mass (ILVM) was within normal ranges for both groups (RA: 70.31 ± 13.21 g/m^2^, control: 72.59 ± 19.28 g/m^2^). These findings emphasize that traditional echocardiography may lack the sensitivity required to identify early cardiac dysfunction in patients with RA.

Several previous studies support these findings. A systematic analysis by Aslam et al. demonstrated that traditional echocardiographic indices, such as LVEF and LV mass, frequently do not detect subclinical left ventricular failure in patients with RA, despite the presence of cardiovascular risk factors (14). This highlights the necessity for more sophisticated diagnostic instruments like STE to identify early indicators of heart dysfunction, particularly in those with chronic inflammatory disorders like RA.

### 5.2 Myocardial strain and subclinical LVSD

This study revealed a significantly reduced GLS in RA patients relative to the control group (18.99 ± 2.81% vs. 20.42 ± 1.33%, *p* = 0.015). Furthermore, one-third of RA patients demonstrated subclinical LVSD (GLS < 18%), whereas no instances were noted in the control group. This finding aligns with previous studies that also showed the capacity of STE to identify subclinical LVSD in RA patients exhibiting normal LVEF (15, 16).

For instance; Ikonomidis et al. (15) were pioneers in investigating cardiac deformation in RA patients, revealing dramatically diminished GLS values correlated with nitro-oxidative stress and endothelial dysfunction (15). Cioffi et al. (16) indicated that diminished GLS and global circumferential strain (GCS) were indicative of impending cardiovascular events in RA patients, demonstrating the prognostic significance of myocardial strain assessments (16). The data indicate that STE is a very sensitive instrument for the early identification of subclinical LVSD, potentially improving cardiovascular outcomes in RA patients by timely detection and management.

### 5.3 Factors associated with subclinical LVSD

Our univariate analysis revealed multiple covariates substantially linked with GLS, including anemia (*r* = −0.368, *p* = 0.027), age (*r* = −0.365, *p* = 0.029), diabetes mellitus (*r* = −0.540, *p* = 0.001), and E/A (*r* = 0.351, *p* = 0.036). Anemia was identified as the most significant independent predictor of subclinical LVSD, with a multivariate odds ratio (OR) of 11.39 (95% CI: 1.57–82.89, *p* = 0.016). This study is among the first that looks into the relationship between anemia and GLS in individuals with RA. Anemia has been demonstrated to adversely affect myocardial strain in several populations, with Zhou et al. indicating a notable decrease in GLS among individuals with diminished hemoglobin levels (17). Our investigation found hemoglobin as the most effective predictor of subclinical LVSD, as demonstrated by the ROC curve analysis (AUC = 0.752, 95% CI: 0.577–0.927, *p* = 0.02).

The association between anemia and reduced myocardial strain in RA patients probably involves multiple processes. Chronic inflammation, typical of RA, facilitates the onset of anemia via increased concentrations of pro-inflammatory cytokines, including TNF-*α* and IL-6. These cytokines do not only inhibit erythropoiesis but also facilitate myocardial fibrosis and diminish myocardial compliance, hence intensifying the impact of anemia on cardiac function (18–20). Consequently, managing anemia in patients with RA may positively impact their hematologic and cardiovascular health.

Besides anemia, diabetes mellitus (DM) was associated with reduced GLS in univariate analysis, however, it lacked significance in multivariate models. Multiple studies have indicated that diabetes mellitus (DM) correlates with myocardial strain impairment in RA patients, with Fine et al. (21) and Cioffi et al. (16). demonstrating that RA patients with DM face an elevated risk of subclinical LVSD (16, 21). The association between diabetes mellitus and GLS impairment is likely multifactorial, involving both systemic inflammation and the microvascular problems typical of diabetes.

### 5.4 Disease activity and immunological profile

Interestingly, our study did not find significant associations between GLS and traditional markers of disease activity, such as DAS28-CRP, nor with immunological markers such as rheumatoid factor (RF) and anti-citrullinated protein antibodies (anti-CCP). The results align with previous investigations conducted by Cioffi et al. (16) and Hanvivadhanakul et al. (22) which similarly did not establish a correlation between GLS and RA disease activity or immunological status (16, 22). This indicates that whereas disease activity significantly contributes to joint inflammation and damage, additional factors, such as persistent systemic inflammation and comorbidities like anemia, may exert a more substantial influence on myocardial function.

## 6 Limitations

While our sample size was adequate to identify statistically significant changes, the comparatively brief duration of RA in certain patients may restrict the generalizability of our results. RA is a progressive condition, and prolonged heart involvement may become more evident with extended disease duration. Subsequent research should encompass patients with a broader spectrum of disease durations to comprehensively assess the cardiovascular ramifications over time.

A major limitation is the absence of potential impacts of disease-modifying antirheumatic medications (DMARDs), especially biologics, on myocardial strain. Research indicates that biologic medicines, including TNF-*α* inhibitors, may mitigate systemic inflammation and confer preventive cardiovascular benefits, however, other studies have expressed concerns regarding neutral or detrimental cardiovascular consequences. Classifying patients according to their usage of DMARDs, particularly biologics may yield a greater understanding of the impact of these drugs on myocardial function in RA.

An additional limitation is the lack of evaluation for coronary microvascular dysfunction, which is increasingly acknowledged as an early factor in myocardial strain abnormalities, especially in RA. Coronary microvascular dysfunction may precede manifest ischemic heart disease and is frequently missed by standard imaging techniques. The integration of modern imaging modalities, such as cardiac magnetic resonance (CMR) and coronary flow reserve (CFR) assessment, will aid in distinguishing the etiologies of subclinical myocardial dysfunction in RA patients, including microvascular involvement.

Ultimately, although patients with recognized cardiovascular disorders were excluded, silent coronary artery disease (CAD) may have been found in certain individuals. Patients with RA are at an elevated risk of developing asymptomatic coronary artery disease due to their chronic inflammatory condition. Echocardiography, although a helpful diagnostic technique, does not yield direct information regarding coronary perfusion. Future research should incorporate more extensive cardiovascular evaluations, including coronary angiography or stress imaging, to exclude the impact of silent CAD on myocardial strain impairment.

## 7 Conclusion

This study confirmed that STE is an effective method for identifying latent LVSD in RA patients who exhibit normal left ventricular function on standard echocardiography. A considerable number of RA patients demonstrated subclinical LVSD, which would have remained undiagnosed without STE. The significant correlation between anemia and diminished myocardial strain indicates that hematologic evaluations, especially hemoglobin concentrations, may enhance the identification of patients at elevated risk for cardiovascular complications. The findings point out the necessity to broaden cardiovascular assessment in RA beyond traditional indicators and echocardiography.

Clinicians can more precisely detect and manage cardiovascular risks in patients with RA by implementing advanced imaging techniques like STE into routine practice, especially in those patients with additional risk factors like diabetes or anemia. Timely identification of subclinical LVSD facilitates preemptive measures to avert the advancement to manifest heart disease, hence enhancing long-term cardiovascular results in patients with RA. This study highlights the necessity of a more thorough and tailored strategy for cardiovascular risk management in RA, with the potential to transform clinical practice towards early and personalized care.

## Data Availability

The raw data supporting the conclusions of this article will be made available by the authors, without undue reservation.
